# High Procalcitonin, C-Reactive Protein, and α-1 Acid Glycoprotein Levels in Whole Blood Samples Could Help Rapid Discrimination of Active Tuberculosis from Latent Tuberculosis Infection and Healthy Individuals

**DOI:** 10.3390/microorganisms10101928

**Published:** 2022-09-28

**Authors:** Yun-Jeong Kang, Heechul Park, Sung-Bae Park, Jiyoung Lee, Hyanglan Hyun, Minju Jung, Eun Ju Lee, Min-A Je, Jungho Kim, Yong Sung Lee, Sunghyun Kim

**Affiliations:** 1 Department of Clinical Laboratory Science, College of Health Sciences, Catholic University of Pusan, Busan 46252, Korea; 2 Department of Laboratory Medicine, Good Samsun Hospital, Busan 47007, Korea; 3 Clinical Trial Specialist Program for In Vitro Diagnostics, Brain Busan 21 Plus Program, Graduate School, Catholic University of Pusan, Busan 46252, Korea; 4 Department of Biomedical Laboratory Science, Masan University, Changwon 51217, Korea; 5 Research Institute of Dream DX Inc., Busan 46252, Korea; 6 Department of Forensic Science, Graduate School, Catholic University of Pusan, Busan 46252, Korea

**Keywords:** active tuberculosis, latent tuberculosis infection, acute phase proteins, diagnostic biomarkers

## Abstract

Tuberculosis (TB) management is important for prompt discrimination of latent TB infection (LTBI) from active TB and proper treatment. Whole blood Interferon-gamma (IFN-γ) release assay (IGRA) is used to diagnose LTBI based on the secretion of IFN-γ by T-cells in the whole blood by using a specific antigen of *Mycobacterium tuberculosis*. However, the ability of IGRA to distinguish active TB from LTBI is considerably limited. Distinguishing active TB from LTBI is necessary to identify indicators that can be used to effectively manage TB and develop diagnostic methods. In the present study, we used a Luminex multiplex bead array (a bead-based antibody–antigen sandwich method). The whole blood level of acute phase proteins (APPs), such as endoglin (ENG), procalcitonin (PCT), C-reactive protein (CRP), and α1-acid glycoprotein (AGP), in active TB, LTBI, and healthy individuals were analyzed and quantified. The APP test results for the serum and whole blood samples showed that the levels of PCT, CRP, and AGP were significantly increased (*p* < 0.0500; area under curve = 0.955) in active TB. The level of these markers in the whole blood of active TB, LTBI, and healthy individuals could provide data for effective diagnosis and treatment of TB.

## 1. Introduction

Tuberculosis (TB), a major infectious disease caused by *Mycobacterium tuberculosis* (MTB), represents a major global public health problem [1]; it is characterized by high infection and mortality rates and is one of the most serious infectious diseases [2]. Although the number of TB cases in the Republic of Korea has been decreasing, the country’s TB incidence and mortality rates in 2016 were 77 and 5.2 per 100,000 people, respectively, which were the highest among the member countries of the Organization for Economic Cooperation and Development (OECD) (Paris, France) [3].

Approximately 5% of people infected with MTB experience active TB for two to five years, and the remaining 95% have latent TB infection (LTBI) [4]. LTBI usually lacks the clinical symptoms of active TB, such as fever, chills, night sweats, weight loss, cough, hemoptysis, and abnormalities found during chest X-ray (CXR) examinations [5]. To date, nearly one-third of the world’s population has LTBI, approximately 10% of whom progress to active TB during their lifetimes [4].

Currently, TB can be diagnosed as LTBI when using the tuberculin skin test (TST) or Interferon-gamma (IFN-γ) release assay (IGRA), along with CXR, physical examination, and assessment of TB exposure and medical history [6]. However, both TST and IGRA tests could not distinguish active TB from LTBI [7]. It is, therefore, necessary to distinguish disease states and to conduct rapid examination. Thus, researchers conducted studies to distinguish LTBI from active TB by using cytokines specific to the MTB antigens, by using differentially expressed genes with subsequent validation via reverse transcription polymerase chain reaction (RT-PCR), and by using monocyte chemoattractant protein-1 (MCP-1) [8,9,10]. 

As MTB enters a host, the pathogenic mechanism between MTB and the host is based on protein expression and on protein–nucleic acid interactions [11]. TB-related proteins can then be used as prognostic and diagnostic markers to distinguish active TB [12]. Technological high-throughput screening has evolved rapidly, and it offers a comprehensive platform for TB research and biomarker discovery [13]. In particular, serum levels of acute phase proteins (APPs) and cytokines distinguish patients with TB. Of these APPs, C-reactive protein (CRP) is often used as a diagnostic marker among children in clinical practice [14]. TB is associated with changes in endogenous protein levels in serum [15]. Acute-phase reaction proteins are produced in the liver in response to inflammation [16]. Endoglin (ENG) is a type I integrated membrane glycoprotein and is commonly known as an angiogenic marker. ENG had an impact on the inflammatory state during inflammatory-related diseases [17]. In addition, one study reported that ENG increases by a factor of 4.9 in iTRAQ and 11.5 in ELISA compared to human immunodeficiency virus (HIV –MTB co-infection [18]. CRP levels increase in response to interleukin (IL)-6-mediated purulent infections, such as active TB [19]. Hepatic α1-acid glycoprotein (AGP) production increases not only in acute inflammation but also in pulmonary TB, and similar results have been found in TB patients with specific glycosylation patterns for serum AGP, which is useful in the discriminative diagnosis of bacterial lung infections [20]. Recent studies have reported a specific correlation between serum procalcitonin (PCT) indices in diagnosing active TB [21]. However, the ability of the current methods to distinguish active TB from LTBI is limited [22,23]. 

To differentiate active TB from LTBI, assistive biomarkers must be developed, and effective TB diagnosis, treatment, and management are needed. In the present study, multiplex bead arrays target serum APP markers, such as ENG, CRP, AGP, and PCT, in order to discriminate the active TB, LTBI, and healthy individuals. A total of 126 whole blood and serum samples were collected and used for the present study. 

## 2. Materials and Methods

### 2.1. Clinical Samples

A total of 126 human whole blood and serum samples were collected from April 2018 to March 2019 at the Department of Laboratory Medicine, Good Samsun Hospital, Busan, the Republic of Korea. This study was approved by the Institutional Review Board (IRB) of the Catholic University of Pusan (IRB Approval No.: CUP IRB-2019-01-010). All enrolled individuals were more than 20 years old. All groups excluded those with HIV infection, diabetes mellitus (DM), cancer, or autoimmune disease, and those who had received chemotherapy within the last 3 months and had a history of active TB treatment. The clinical characteristics of the present study are shown in Table 1. The active TB group was confirmed positive for Ziehl–Neelsen AFB stain, mycobacterial culture, MTB-PCR using respiratory specimens, and CXR. AFB stain results were established according to CDC guidelines [24]. The LTBI group was confirmed positive for whole blood IGRA, the QuantiFERON TB-Gold in-tube (QFT-GIT) test, and negative for active TB diagnostic assay; it also had no active TB symptoms. The healthy individuals were confirmed negative for whole-blood IGRA and CXR examination, and had no active TB symptoms.

### 2.2. Whole Blood Collection and Serum Preparation

Whole blood samples were collected using VACUETTE^®^ EDTA blood collection tubes (Greiner Bio-One, Frickenhausen, Austria) containing EDTA anticoagulant. Samples were centrifuged at 4000× *g* for 15 min to obtain the serum, which was stored at −20 °C in a 1.5 mL microcentrifuge tube until use.

### 2.3. Analysis of Serum Acute Phase Protein Markers

Peripheral venous whole blood samples were used to analyze the biomarkers of interest via magnetic Luminex multiplex bead array by using Luminex^®^ 100/200™, Human Premixed Multi-Analyte kit (R&D Systems, Minneapolis, MN, USA) and Human Cardiovascular Disease Magnetic Bead Panel 3 MILLIPLEX^®^ MAP kit (EMD Millipore Corporation, Billerica, MA, USA) according to the manufacturers’ instructions. The PCT and ENG were analyzed and quantified with the Human Premixed Multi-Analyte kit (R&D Systems), whereas the CRP and AGP were analyzed and quantified with the Human Cardiovascular Disease Magnetic Bead Panel 3 MILLIPLEX^®^ MAP kit (EMD Millipore Corporation). The samples were evaluated using a MAGPIX^®^ multiplexing system (Luminex Corporation, Austin, TX, USA), which is a fluorescent bead-based instrument. Results were analyzed and interpreted using the Luminex xPONENT^®^ software (Luminex Corporation).

### 2.4. Statistical Analysis

Statistical analysis was performed using the GraphPad Prism 5.0 software (GraphPad Software, San Diego, CA, USA). Differences in APP markers among the active TB, LTBI, and healthy individuals were analyzed, and 95% confidential intervals were calculated. An unpaired *t*-test was performed to compare the three groups. Additionally, a receiver operator characteristic (ROC) curve analysis was conducted to confirm the clinical usefulness of the results and to determine the cut-off value, specificity, and sensitivity of the assays [25]. The *p* values of <0.05 were considered statistically significant.

## 3. Results

### 3.1. Quantitative APP Marker Analysis Results for the Active TB, LTBI, and Healthy Individuals

**Figure 1:**
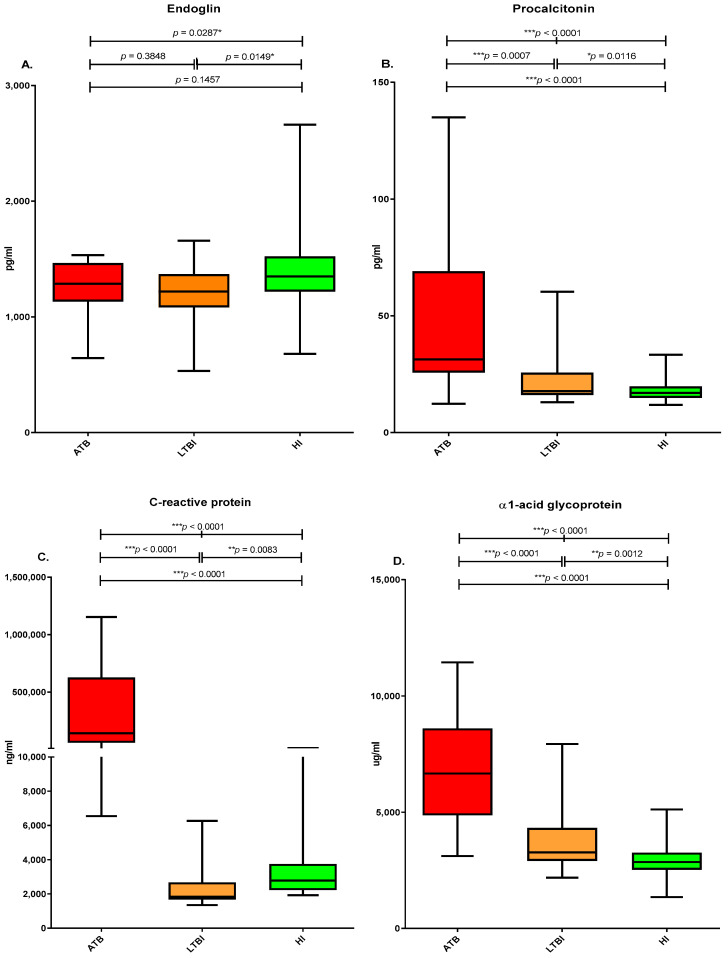
Comparison of acute-phase proteins between the active tuberculosis (ATB; Red), Latent tuberculosis infection (LTBI; Yellow), and healthy individuals (HI; Green). (**A**) Endoglin (ENG); (**B**) procalcitonin (PCT); (**C**) C-reactive protein (CRP); (**D**) α1-acid glycoprotein (AGP). * *p* < 0.05, ** *p* < 0.001, *** *p* < 0.0001.

Based on the APPs data analysis, the mean ENG values for the active TB, LTBI, and healthy individuals were 1267.88 ± 214.47, 1209.12 ± 252.60, and 1371.81 ± 303.69 pg/mL, respectively, and their corresponding mean PCT values were 44.11 ± 29.21, 22.68 ± 11.67, and 18.15 ± 4.58 pg/mL. The mean CRP values for the active TB, LTBI, and healthy individuals were 343,491.91 ± 362,153.63, 2358.38 ± 1213.21, and 3375.52 ± 1833.75 ng/mL, respectively; their corresponding AGP values were 6886.68 ± 2438.14, 3749.57 ± 1369.43, and 2969.90 ± 795.71 µg/mL. Compared with the LTBI and healthy individuals, the active TB group had significantly higher mean PCT, CRP, and AGP. Compared with the healthy groups the LTBI group had significantly higher mean PCT, CRP, and AGP (Figure 1, Table 2). The active TB and LTBI groups significantly differed in terms of PCT (*p* = 0.0007) and in terms of CRP and AGP. (*p* < 0.0001). The active TB and healthy individuals significantly differed in terms of ENG (*p* = 0.0149), PCT (*p* = 0.0112), CRP (*p* = 0.0083), and AGP (*p* = 0.0012). Moreover, the active TB and healthy individuals significantly differed in terms of PCT, CRP, and AGP (*p* < 0.0001). The active TB, LTBI, and healthy individuals significantly differed in terms of ENG (*p* = 0.0287) and in terms of PCT, CRP, and AGP (*p* < 0.0001) (Table 3).

### 3.2. ROC Curve Analysis Based on the Results for APPs

**Figure 2:**
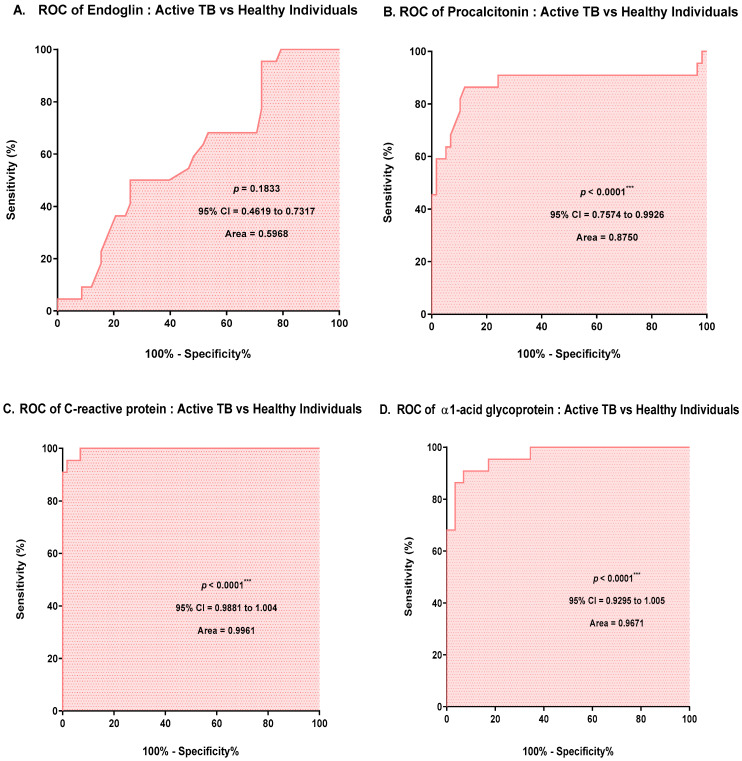
ROC curve analysis of acute-phase proteins between the active TB and healthy individuals. (**A**) Endoglin (ENG); (**B**) procalcitonin (PCT); (**C**) C-reactive protein (CRP); (**D**) α1-acid glycoprotein (AGP). *** *p* < 0.0001.

ROC curve analysis was performed to ensure that the results were clinically applicable. The *p* value of the ROC curve and the AUC for the APPs were as follows: for PCT, *p* < 0.0001 and AUC = 0.8750; for CRP, *p* < 0.0001 and AUC = 0.9961; and for AGP, *p* < 0.0001 and AUC = 0.9671. No significant differences in the ROC curves of ENG were observed (Figure 2). The p values for PCT, CRP, and AGP were all statistically significant (*p* < 0.0500), and the AUC was approximately 0.9550 (Figure 2).

### 3.3. Diagnostic Performance of the Quantitative APP Markers

To investigate the clinical relevance of the APP markers, the AUC, sensitivity, and specificity of ENG, PCT, CRP, and AGP were analyzed (Table 4). All APP markers except endoglin had AUC values of 0.87 or higher, indicating good diagnostic performance in ATB (*p* < 0.001). In particular, CRP showed high sensitivity of 95.45% and specificity of 98.28%, and AGP showed high sensitivity of 90.91% and specificity of 93.10%.

## 4. Discussion

TB is a serious infectious disease with high infection and mortality rates [2]. According to the 2021 WHO report, approximately half a million (range, 417,000–556,000) new cases of rifampicin-resistant TB (of which 78% are multi-drug-resistant TB) have been diagnosed, and this phenomenon is a major concern [26]. Among the member countries of the OECD, the Republic of Korea has high prevalence and mortality rates for TB [3].

TB management is important for the rapid differentiation of LTBI from active TB and for the identification of the appropriate anti-TB treatment. Currently, either the TST or IGRA test is used to diagnose LTBI [27]. A single test can erroneously diagnose LTBI and active TB [7]. Although numerous studies have been conducted to distinguish LTBI from active TB, according to a China-based study, the ENG levels of the HIV–MTB coinfection group showed a 4.9-fold increase in iTRAQ proteomics, and an 11.5-fold increase in enzyme-linked immunosorbent assay (ELISA) results compared with those of the HIV-free TB group [18].

ENG is an important glycoprotein lipid involved in extracellular matrix (ECM) synthesis, angiogenesis, and cell proliferation, and is expressed by endothelial cells activated through the transforming growth factor-β (TGF-β) pathway [28,29]. ENG is overexpressed in hepatocellular carcinoma (HCC) micro vessels because HCC is characterized by neovascularization by tumor cells [30,31]. ENG expression is correlated with tumor progression and microvascular density, such as colon cancer, lung cancer, and prostate cancer, and is a key protein for tumor proliferation and metastasis [32]. In the present study, there were no tumor patients as an underlying disease in the ATB and LTBI; ENG levels in the active TB group were not significantly different (*p* > 0.0500, AUC = 0.5968) from those in the LTBI group and healthy individuals. AGP is one type of immunomodulatory substance; it forms granulomas when macrophages engulf the pulmonary TB pathogen, and it is produced in the extracellular matrix [33]. During MTB infection, AGP is produced in the lungs, and macrophages are an important source [20]. AGP is a major APP produced by the liver, and its level increases with systemic damage, inflammation, or infection [34,35]. One study has identified AGPs in serum with a sensitivity of 81.2% (69/85) and a specificity of 95.2% (80/84) via ELISA. In addition, a sensitivity of 81.2% (69/85) and a specificity of 90.1% (64/81) differentiated the active TB from the healthy individuals [13]. Indeed, in this study, the AGP values of the active TB group obtained using the magnetic Luminex multiplex bead array were significantly higher than those of the LTBI and healthy individuals (*p* < 0.0001, AUC = 0.9671). Research has suggested that CRP is an acute inflammatory reactant whose levels increase in response to IL-6-mediated purulent infections, such as active TB [19]. Indeed, our data showed that the CRP value in the active TB group significantly increased (*p* < 0.0001, AUC = 0.9961) compared with those in the LTBI group and healthy individuals. As a biomarker, PCT is a propeptide of calcitonin with no hormonal activity; it is a biomarker of the systemic inflammatory response to bacterial infection that is not significantly age related [36,37]. In a study that evaluated the usefulness of PCT for the differentiation between pulmonary TB and other lung infections, the sensitivity and specificity of PCT for differentiating TB and non-TB groups were 42% (95% CI = 30–56) and 87% (95% CI = 63–96), respectively. In the present study, the PCT levels of the active TB group was significantly higher (*p* < 0.0010, AUC = 0.8750) than those of the LTBI group and healthy individuals.

Despite numerous research projects that have been conducted, a gold standard that could differentiate active TB and LTBI groups remains inexistent. This study thus aimed to compare the APP levels in serum samples to differentiate active TB and LTBI groups and ultimately identify biomarkers that can be used to differentiate LTBI from active TB. In conclusion, PCT, CRP, and AGP are biomarkers that could differentiate the active TB, LTBI, and healthy individuals at a statistically significant level. However, additional screening tests, such as CBC and WBC differential counts, and additional acute inflammatory mediator tests, are expected to improve the quality of TB diagnosis. The whole blood levels of these markers of active TB, LTBI, and healthy individuals are useful as indicators for differential diagnosis and as basic data for effective diagnosis and treatment. 

This study has several limitations. First, the sample size was small. The expression of target molecules should be further investigated in a larger number of patients from multiple centers. Second, there is some age disparity between study groups. CRP that has a correlation with the level of expression according to age require further study in similar age groups. Further studies involving a larger number of clinical samples and a larger population are needed to validate the importance of this study and to improve the accuracy of discrimination between active TB and LTBI groups.

## 5. Conclusions

We suggest that PCT, CRP, and AGP are potential biomarkers that could differentiate the active TB, LTBI, and healthy individuals at a statistically significant level.

## Figures and Tables

**Table 1:** Demographic and clinical characteristics of study subjects.

Demographic and Clinical Characteristics	Active TB	LTBI	Healthy Individuals
Total number (*n* = 129)	22	29	58
Median age (range), years	55.2 (23–89)	44.6 (21–70)	33.2 (22–61)
Gender, male/female	15/7	6/23	12/46
AFB stain results			
+ positive, *n* (%)	2 (9.1)	NA	NA
++ positive, *n* (%)	4 (18.2)	NA	NA
+++ positive, *n* (%)	4 (18.2)	NA	NA
++++ positive, *n* (%)	4 (18.2)	NA	NA
Negative	8 (36.4)	NA	NA
AFB culture results			
Positive, *n* (%)	19 (86.4)	NA	NA
Negative, *n* (%)	3 (13.6)	NA	NA
MTB-PCR results			
Positive, *n* (%)	21 (95.5)	NA	NA
Negative, *n* (%)	1 (4.5)	NA	NA
CXR			
Positive, *n* (%)	22 (100.0)	4 (13.8)	0 (0.0)
Negative, *n* (%)	0 (0.0)	25 (86.2)	58 (100.0)
IGRA test results			
Positive, *n* (%)	NA	29 (100.0)	0 (0.0)
Negative, *n* (%)	NA	0 (0.0)	58 (100.0)

General characteristics of the groups involved in the study showing the number of subjects per group (n), the mean age, gender, AFB stain results; + positive: rare; ++ positive: few; +++ positive: moderate; ++++ positive: many; Negative: AFB not found, MTB-PCR results, chest X-ray (CXR), IGRA test results; *n*: number; healthy individuals: non-infected healthy group; LTBI: latent tuberculosis infection group; active TB: active pulmonary tuberculosis group, NA: not applied.

**Table 2:** Acute-phase protein levels in serum samples between active TB, LTBI, and healthy individuals.

Acute-Phase Protein Markers	Active TB, Mean Level ± SD	LTBI, Mean Level ± SD	Healthy Individuals, Mean Level ± SD
Endoglin (pg/mL)	1267.88 ± 214.47	1209 ± 252.60	1371.81 ± 303.69
Procalcitonin (pg/mL)	44.11 ± 29.21	22.68 ± 11.67	18.15 ± 4.58
C-reactive protein (ng/mL)	343,491.91 ± 362,153.63	2358.38 ± 1213.21	3375.52 ± 1833.75
α1-acid glycoprotein (µg/mL)	6886.68 ± 2438.14	3749.57 ± 1369.43	2969.90 ± 795.71

Abbreviations: LTBI, latent tuberculosis infection; SD, standard deviation.

**Table 3:** Statistical data of quantitative acute-phase protein analysis between the active TB, LTBI, and healthy individuals.

Acute-Phase Protein Markers	Active TB vs. LTBI	LTBI vs. Healthy Control	Active TB vs. Healthy Individuals	Active TB vs. LTBI vs. Healthy Individuals
Endoglin	0.3848	0.0149 *	0.1457	0.0287 *
Procalcitonin	0.0007 ***	0.0112 *	<0.0001 ***	<0.0001 ***
C-reactive protein	<0.0001 ***	0.0083 **	<0.0001 ***	<0.0001 ***
α1-acid glycoprotein	< 0.0001 ***	0.0012 **	<0.0001 ***	<0.0001 ***

* *p* < 0.05, ** *p* < 0.001, *** *p* < 0.0001.

**Table 4:** The diagnostic utility of acute-phase protein markers for tuberculosis.

Acute-Phase Protein Markers	AUC (95% CI)	Cut-Off Value	Sensitivity (%) (95% CI)	Specificity (%) (95% CI)	*p* Value
Endoglin	0.60 (0.46–0.73)	>1330 ng/mL	54.55 (32.21–75.61)	53.45 (39.87–66.66)	0.1833
Procalcitonin	0.87 (0.76–0.99)	>23 ng/mL	86.36 (65.09–97.09)	87.93 (76.70–95.01)	<0.0001
C-reactive protein	0.99 (0.99–1.00)	>8853 ng/mL	95.45 (77.16–99.88)	98.28 (90.76–99.96)	<0.0001
α-1-acid glycoprotein	0.98 (0.93–1.00)	>4548 ng/mL	90.91 (70.84–98.99)	93.10 (83.27–98.09)	<0.0001

Abbreviations: AUC, area under the receiver operating characteristic curve; CI, confidence interval.

## Data Availability

The data generated or analyzed during this study are included in this published article and its additional files. Some of the datasets are available from the corresponding author upon reasonable request.
